# Temporal changes in diet quality and the associated economic burden in Canada

**DOI:** 10.1371/journal.pone.0206877

**Published:** 2018-11-08

**Authors:** Léon Nshimyumukiza, Jessica R. L. Lieffers, John Paul Ekwaru, Arto Ohinmaa, Paul J. Veugelers

**Affiliations:** 1 Population Health Intervention Research Unit, School of Public Health, University of Alberta, Edmonton, Alberta, Canada; 2 Department of Family and Emergency Medicine, Faculty of Medicine, Université Laval, Quebec, Quebec, Canada; 3 College of Pharmacy and Nutrition, University of Saskatchewan, Saskatoon, Saskatchewan, Canada; McMaster University, CANADA

## Abstract

A high-quality diet is associated with a reduced of risk of chronic disease and all-cause mortality. In this study, we assessed changes in diet quality and the associated economic burden in the Canadian population between 2004 and 2015. We used a prevalence-based cost-of-illness approach. We first calculated the diet quality using the Healthy Eating Index-Canada-2010 (HEI-C-2010) and 24-hour recall data from the Canadian Community Health Surveys (CCHS) on nutrition (CCHS 2004 cycle 2.2 and the CCHS-NU 2015). We then retrieved relative risks of HEI-2010 quintiles for chronic diseases from meta-analyses. Based on the proportions of the population following diets of varying qualities and these relative risks, we computed the population-attributable fractions and attributable costs (direct health care and indirect costs) by survey year (2004 and 2015) as well as by age and sex group. Costs were estimated in 2017 Canadian dollars for comparison purposes. We observed that on average the diet quality of Canadians improved between 2004 and 2015: the proportion of the Canadian population that did not eat a diet of high quality decreased from 83% to 76%. This improvement in diet quality translated in a decrease in economic burden of $133 million, down from $13.21 billion in 2004 to $13.08 billion in 2015. The economic burden decreased by $219 million among males but increased by $86 million among females. It also decreased among people under the age of 65 years ($333 million) but increased among those over 65 years ($ 200 million). Our findings suggest that, despite some temporal improvements, the diet of the majority of Canadians is of poor quality resulting in a high attributable economic burden. Policy and decision makers are encouraged to expand nutrition programs and policies and to specifically target the elderly in order to prevent chronic diseases and reduce health care costs.

## 1. Introduction

Poor diet quality is a major, though modifiable cause of chronic diseases such as cardiovascular diseases, type 2 diabetes, and cancer [1]. In 2015, it was estimated that low intakes of vegetables and fruit, and high intakes of sugar-sweetened beverages, processed foods and sodium accounted for 37% of all deaths worldwide [2]. In Canada, poor diet quality was ranked as the second highest contributor to the burden of chronic diseases and deaths with only smoking ranking higher [3]. Compared to other modifiable chronic disease risk factors (e.g., smoking, alcohol, physical inactivity), diet is especially complex because different combinations of dietary components that are both protective and harmful are consumed together which can act either synergistically or antagonistically [4]. Therefore, when quantifying the impact of diet on chronic disease outcomes, focusing on the overall quality of the diet rather than on single foods or nutrients is a valid strategy. Published studies have shown that diets which score high on diet quality indices, such as Health Eating Index (HEI), are associated with a lower risk of chronic diseases and a lower risk of death [5, 6].

There are two main *a priori* diet quality indices based on Dietary Guidelines commonly used in North America: the Healthy Eating Index (HEI) and the Alternative Healthy Eating Index (AHEI). The HEI was first developed in 1995 to measure compliance with the Dietary Guidelines for Americans (DGAs) [7]. It was subsequently updated three times to reflect the 2005 DGAs (i.e. HEI-2005) [8], the 2010 DGAs (i.e. HEI-2010) [9] and the 2015 DGAs (i.e. HEI-2015) [10, 11]. HEI scores range between 0–100 and are based on amounts of 12 components (foods and nutrients) consumed per 1,000 kcal energy intake. The first AHEI was developed in 2002 [12] and subsequently updated in 2012 [13]. The AHEI was developed as an alternative to the original HEI to better predict chronic disease reduction [12]. The AHEI uses an absolute intake approach instead of a foods and nutrients density one used by HEI [5]. The AHEI has a maximum of 110 points and consists of 11 components (10 points maximum each). Recent meta-analyses found that the more recent diet quality indexes versions of both HEI and AHEI (i.e. HEI-2010, AHEI-2010) were similarly associated with a reduced risk of chronic diseases and associated mortality [5, 6].

In Canada, Garriguet et al. 2009 [14] adapted the 2005 American version of the HEI (HEI-2005) to conform to Canadian recommendations in the 2007 Canada’s Food Guide (CFG) [15] and Jessri et al (2017) [16] adapted the American HEI-2010 version to the 2007 CFG (HEI-C 2010). To our knowledge, no adaptation of the AHEI nor the HEI-2015 to the Canadian context was performed to date. Cost of illness studies are a resource used by policy and decision makers to help understand the economic burden of chronic disease associated with different risk factors [17]. However, few studies have estimated the economic burden of overall diet quality but instead have focused on single foods or nutrients [18–23]. Of the studies that have focused on diet quality, a few different approaches have been used. For example, Scarborough et al. (2011) [24] in the United Kingdom (UK), estimated that the direct National Health Service (NHS) costs associated with poor diets were £5.8 billion from the 2006–2007 financial year assuming that 33% of chronic diseases are attributable to poor diet. Candari et al. (2017) [25] took a different approach by using the Alternative Healthy Eating Index (AHEI) [12] and focusing on the economic burden of unhealthy diets specifically for one disease (type 2 diabetes). These authors, using an incidence-based approach (which estimates the lifetime costs of a condition from its onset until its disappearance), projected that the total economic burden of unhealthy diets will be € 883 million in 2020 for five European countries (France, Germany, Italy, Spain, and UK). Recently, Lieffers et al. (2018) [26] reported that not meeting Canadian recommendations for eight different foods was responsible for CAD$13.8 billion/year in direct health care and indirect costs in Canada [26]. This estimation was conducted using information on dietary intakes from the 2004 Canadian Community Health Survey (CCHS cycle 2.2) [27], and information on costs from the Economic Burden of Illness resource, and the National Health Expenditure Trends.

Given a growing interest in focusing on overall diet quality [28], this study assessed changes in diet quality and the associated economic burden in the Canadian population between 2004 and 2015. Unhealthy eating was defined as not consuming a diet of high quality as assessed by the HEI-2010 adapted to the Canadian population (HEI-C 2010) [16]. The HEI-C 2010 was chosen because it is the most recent adaptation of the HEI (i.e. HEI-2010) to the Canadian context and is a validated tool (the multidimensionality was confirmed by principal component analysis and the internal reliability was demonstrated i.e. Cronbach’s α = 0.78).

## 2. Methods

We estimated the economic burden of unhealthy eating using a prevalence approach based on population-attributable fractions (PAF) [17, 29] in the perspective of Canadian society for the year 2017. We followed four steps to complete our calculations: 1) estimation of proportions of the Canadian population in specific age and sex-categories who consumed different diet qualities based on the HEI-C 2010 criteria in 2004 and 2015; 2) extraction of relative risks for chronic diseases by HEI quintile category; 3) calculation of population-attributable fractions (PAFs) for year 2004 and 2015 for each age and sex category, 4) estimation of direct health care and indirect costs attributable to low HEI scores and calculation of difference in costs between 2004 and 2015 using 2017 Canadian dollar values.

To estimate the economic burden of unhealthy eating in Canada, 3 data sources were used: 1) Statistics Canada: to retrieve data on dietary intakes from the 2004 and 2015 CCHS nutrition surveys [27, 30]; 2) Meta-analyses [5, 6]: to retrieve relative risks about HEI scores and chronic diseases, and 3) Administrative data to calculate direct and indirect costs i.e. Economic Burden of Illness in Canada (EBIC) online tool from the Public Health Agency of Canada (PHAC) [31] and 2017 National Health Expenditure Trends report from the Canadian Institute for Health Information (CIHI) [32])

The University of Alberta Research Ethics Board has approved this study (Approval no: Pro00073196). Data from the 2004 and 2015 Canadian Community Health Surveys were accessed from Statistics Canada through the Research Data Centers Program (RDC).

### Estimation of the distribution of diet quality in the Canadian population

To estimate the distribution of diet quality in Canadian population, we used data on dietary intakes from the 2004 and 2015 CCHS nutrition surveys which were collected using 24-hour dietary recalls administered using a computerized automated Multiple Pass Method [33]. 35,107, and 20,487 respondents, respectively in 2004 and in 2015 completed a 24-hour dietary recall; of those respondents, 10,786 (30.7) and 7,623 (37%), respectively in 2004 and in 2015, completed and a second recall. Details regarding sampling design and data collection are available elsewhere [27, 30]. We excluded from our study respondents who were < 2 years of age, pregnant women, children consuming only breast milk, and respondents who did not have valid 24-hour recalls. For the present analysis, we considered the 24-hour recalls from 33,932 and 19,797 respondents from 2004 and 2015, respectively.

We used the HEI-C 2010 criteria to determine the quality of the diets. The HEI-C 2010 has 11 dietary components (8 for adequacy and 3 for moderation). The total scores for HEI-C 2010 range from 0 to 100, a high score representing a diet of high quality. Details for HEI-C 2010 are presented in Table 1.

**Table 1:** Healthy Eating Index-Canada 2010 (HEI-C 2010).

Component	Max pts	Standard for max score	Standard for min score (0)
Adequacy Sub-score	60		
Total fruits and vegetables	10	4–10 servings	No servings
Whole fruit	5	0.84–2.1 servings	No servings
Greens and beans	5	0.42–1.05 servings	No servings
Whole grains	10	1.5–4 servings	No servings
Dairy	10	2–4 servings	No servings
Total protein foods	5	1–3 servings	No servings
Seafood and plant proteins	5	0.32–0.96 servings	No servings
Fatty acids	10	(PUFA^1^ + MUFA^2^)/SFA^3^ ≥ 2.5	(PUFA + MUFA)/SFA ≤ 1.2
Moderation Sub-score	40		
Refined grains	10	<50% of grains refined	≥50% of grains refined
Sodium	8 to 10	AI^4^ to UL^5^	2x UL
Empty calories	20	≤ 19% of energy	≥ 50% of energy
Total HEI-C 2010 Score	100		

Source: Jessri et al.2017 [16]

^1^PUFA: polyunsaturated fatty acids;

^2^MUFA: monounsaturated fatty acids;

^3^SFA: saturated fat;

^4^AI: Adequate intake;

^5^ UL: Tolerable upper intake level.

We used the “Simple Scoring Algorithm” which is the available analytic method to relate the HEI total scores to an outcome as suggested by the National Cancer Institute (NCI) [34]. This Simple Scoring Algorithm calculates the mean of the component scores and total scores across individuals by summing scores across all days per person where more than one 24-hour dietary recall per person is available in the 2004 and 2015 surveys [34]. We adapted the SAS macros, available online [35], for estimating the HEI-2010 scores to the HEI-C 2010 [16] in order to derive the HEI-C 2010 total scores for the Canadian population. Those HEI-C 2010 total scores were categorized in quintiles. By doing so, we aligned with the meta-analyses by Schwingshackl et al. [5, 6] and all studies included that have operationalized HEI-scores in quintiles. Respondents in the highest quintile (quintile 5) were considered to consume a diet of high quality. We considered individuals in the highest quintile as having a healthy diet to maintain the consistency with meta-analyses that have reported the relative risks comparing individuals in the highest quintile (Q5) to those in the lowest (Q1) [5, 6]. We then calculated the relative risks for other quintiles assuming a linear dose-response relationship. This assumption was applied by Krueger et al. (2017) [23] to estimate the relative risks of other categories using the relative risks expressed in terms of highest (quintile 5) versus the lowest one (quintile 1). We calculated HEI-C 2010 scores combining males and females for age groups 2–3 years, 4–8 years, 9–13 years, and sex-specific scores for age-groups 14–18 years, 19–50 years, and ≥51 years, according to Canada’s Food Guide [15]. All statistical analyses were performed using SAS (version 9.4; SAS institute Inc. Cary, NC, USA). To ensure the estimated proportions represent the Canadian population, we used the SAS SURVEYFREQ procedure [36]. We applied population survey weights provided by Statistics Canada and used the balanced repeated replication (BRR) with 500 replicates to take into account for the complex sampling designs used in 2004 and 2015 CCHS [37].

### Relative risks of unhealthy eating according to HEI 2010

We used data from published meta-analyses [5, 6] to retrieve relative risks of unhealthy eating as measured by HEI-2010 for chronic diseases. Based on these sources, we included cardiovascular diseases (ischemic heart disease, ischemic stroke and heart failure); type 2 diabetes and cancers (colorectal, esophagus, stomach/gastric, hepatocellular carcinoma, larynx, oral, lung, pancreas, prostate). In cases where the meta-analyses did not report relative risk estimates for HEI-2010, we used estimates for HEI-2005. Relative risks used in analyses are presented in S1 Table.

### Estimation of population attributable fractions (PAFs)

The PAFs were calculated using the age and sex distributions of unhealthy eating based on HEI-C 2010 scores and the relative risks for chronic diseases mentioned above. The PAF represents the proportion of disease that could be theoretically reduced if the entire Canadian population would consume a high-quality diet (i.e., HEI-C 2010 scores in quintile 5). We used the method recommended by Krueger et al. (2013) [17] to take into account multiple risk exposure levels in the following equation:

$$PAF=\frac{\sum_{i=1}^{n}P_{i}({RR}_{i}-1)}{1+\sum_{i=1}^{n}P_{i}({RR}_{i}-1)}$$

Where P_i_ is the proportion of people in quintile i,

i is the specific quintile of HEI-C 2010 score,

RR is the relative risk for each increase in quintile of HEI-C 2010 score,

RR_i_ = RR ^(Xi-L)^ is the relative risk for quintile i relative to quintile 5 (considered as reference),

X_i_ is the mid value of quintile i

L is the cut-point of quintile 5,

n = 4, the number of quintiles below quintile 5.

We estimated PAFs for both surveys (2004 and 2015) and we calculated the difference in PAFs between year 2015 and 2004 as PAFs_2015_ minus PAFs _2004_. We considered relative risks as random parameters lognormally distributed [38] and we estimated variance from their reported 95% confidence intervals (CI). Based on that, we performed 50,000 Markov Chain Monte Carlo simulations [39] to produce PAFs estimates and their 95% CI.

### Estimation of direct health care and indirect costs attributable to unhealthy eating and changes between 2004 and 2015

We estimated direct health care and indirect costs attributable to unhealthy eating for each disease of interest using the calculated PAFs and direct health care and indirect costs for each age/sex category determined using administrative data (i.e. Economic Burden of Illness in Canada (EBIC) online tool from the Public Health Agency of Canada (PHAC) [31], 2017 National Health Expenditure Trends report from the Canadian Institute for Health Information (CIHI) [32]). First, we calculated the proportions of each direct health care cost component (hospitals, physicians, and drugs) associated with each disease using the 2008 EBIC [31] by age (≤ 14 years, 15–34 years, 35–54 years, 55–64 years, 65–74 years, and ≥75 years) and sex groups. Given that the EBIC provides combined direct health care costs for all types of diabetes combined, a proportion of 0.96 was applied to estimate the proportion of these costs attributable to type 2 diabetes [40]. Second, we multiplied these proportions by the total direct health care costs from the 2017 National Health Expenditure Trends [32] to estimate the direct health care costs for each disease by age and sex group which assumes these proportions remain stable over time. Direct health care costs are presented in S2 Table. Third, we estimated indirect costs using a human capital approach, which was applied in the EBIC 1998 [41]. Using results from EBIC 1998, we calculated ratios of indirect costs (i.e., costs associated with mortality and short and long-term disability) vs direct health care costs for each disease category. Those ratios are presented in S3 Table. These ratios were then multiplied by the calculated 2017 direct health care costs (hospital, physician and drugs) for each disease of interest to obtain indirect costs for each disease for each age and sex group, assuming again that those ratios remain unchanged over time. Fourth, the 2017 direct health care and indirect costs estimated for each disease of interest by age and sex group were multiplied by relevant PAFs (PAFs_2004_ and PAFs_2015_) to determine the economic burden attributable to unhealthy eating estimated in 2017 Canadian dollars.

Changes in economic burden between 2004 and 2015 were considered as differences in 2017 costs between 2015 and 2004 surveys.

### Sensitivity analysis

We performed a sensitivity analysis by re-calculating all PAFs using low and upper boundary estimates of 95% confidence interval of all relative risks considered which were then multiplied by the 2017 direct and indirect costs estimated for each disease of interest by age and sex group.

## 3. Results

Table 2 presents the percentage of the Canadian population by sex and age group falling into the HEI quintiles for the survey years 2004 and 2015. With the exception of age group 55–64 years, the percentage of females reporting a healthy diet (those in quintile 5) increased and the percentage reporting an unhealthy diet (those in quintiles 1, 2, 3, and 4) mostly decreased. With the exception of age group 65–74 years, the percentage of males reporting a healthy diet increased and the percentage reporting an unhealthy diet mostly decreased. On average, the percentage of Canadians with a healthy diet (quintile 5) increased from 17% in 2004 to 24% in 2015, and thus the percentage with an unhealthy diet decreased from 83% in 2004 to 76% in 2015. When comparing HEI-C 2010 mean scores adjusted by quintiles scores, we observed a significant change between 2004 and 2015 in the means of quintile 5. The HEI-C mean score of quintile 5 was significantly higher in 2015 compared to 2004 (see S4 Table).

**Table 2:** Percentages of the 2004 and 2015 Canadian population ≥ 2 years by age and sex and by HEI-C 2010 quintile.

		Female			Male		
		2004	2015	2015 vs 2004	2004	2015	2015 vs 2004
<15 years	Quintile 1	15.7	11.2	-4.5	18.8	12.4	-6.4
	Quintile 2	20.0	15.4	-4.6	18.6	18.2	-0.6
	Quintile 3	20.5	20.8	-0.3	20.3	18.4	-1.9
	Quintile 4	25.2	23.0	-2.2	21.9	22.8	-0.9
	Quintile 5	18.6	29.7	+11.1	20.3	28.1	+7.8
15–34 years	Quintile 1	21.8	18.8	-3.0	34.9	29.9	-4.0
	Quintile 2	24.0	18.4	-5.6	26.8	19.5	-7.3
	Quintile 3	19.6	20.1	+0.5	19.4	24.5	+4.9
	Quintile 4	19.8	19.2	-0.6	13.5	14.6	+0.9
	Quintile 5	14.9	23.4	+8.5	5.4	11.5	+6.1
35–54 years	Quintile 1	14.5	13.9	-0.6	26.1	22.8	-4.7
	Quintile 2	18.5	15.9	-3.4	27.1	20.7	-6.4
	Quintile 3	20.9	17.4	-3.5	20.5	21.7	+1.2
	Quintile 4	22.3	22.2	-0.1	17.1	16.1	-1.0
	Quintile 5	23.7	30.6	+6.9	9.2	18.6	+9.4
55–64 years	Quintile 1	12.5	14.8	+2.3	19.7	18.0	-1.7
	Quintile 2	15.4	12.2	-3.2	24.0	18.6	-5.4
	Quintile 3	17.8	17.4	-0.4	18.0	20.0	+2.0
	Quintile 4	21.7	25.2	+3.5	20.7	19.8	-0.9
	Quintile 5	32.6	30.4	-2.2	17.6	23.6	+6.0
65–74 years	Quintile 1	11.0	11.2	-0.2	18.2	18.4	+0.2
	Quintile 2	17.3	14.9	-2.4	17.3	16.8	-0.5
	Quintile 3	19.4	16.6	-2.6	19.8	28.6	+8.8
	Quintile 4	22.7	22.5	-0.2	23.9	19.8	-4.1
	Quintile 5	29.6	34.8	+5.2	20.8	16.4	-4.4
75+ years	Quintile 1	7.7	12.3	+5.6	15.0	15.9	+0.9
	Quintile 2	15.3	14.5	-0.8	17.7	16.4	-1.3
	Quintile 3	20.4	20.6	+0.2	20.7	20.9	+0.2
	Quintile 4	27.3	18.7	-8.6	23.0	23.2	+0.2
	Quintile 5	29.3	33.9	+4.6	16.5	23.7	+7.2

PAFs estimates for each chronic disease by age and sex group are presented in Table 3. Between 2004 and 2015, PAFs values decreased in all age and sex groups, except for males aged 65–74 years and females aged 55–64 years and ≥75 years.

**Table 3:** Population attributable fractions (PAFs) for unhealthy diet presented by age group, sex and chronic disease.

Age group	Chronic disease (ICD-10 Code)*	PAFs (%)					
		Male			Female		
		2004	2015	2015 vs 2004	2004	2015	2015 vs 2004
< 15 years	Colorectal cancer(C20)	36.7	32.1	-4.6	35.6	30.6	-5.0
	Esophagus cancer(C15)	52.9	48.3	-4.6	51.7	46.7	-5.0
	Stomach/gastric cancer(C16)	19.1	16.5	-2.6	18.5	15.7	-2.8
	Hepatocellular cancer(C22)	44.3	39.8	-4.4	43.1	38.3	-4.8
	Larynx cancer(C32)	59.3	55.0	-4.3	58.1	53.4	-4.7
	Oral cancer(C00-C14)	59.3	55.0	-4.3	58.1	53.4	-4.7
	Pancreas cancer(C25)	23.9	20.7	-3.2	23.1	19.6	-3.5
	Prostate cancer(C61)	10.9	9.2	-1.7	-	-	-
	Lung cancer (C34)	25.4	21.8	-3.6	24.5	20.7	-3.8
	Type 2 diabetes (E10-E14)	20.5	17.6	-2.9	19.8	16.7	-3.2
	Ischemic stroke(I63)	33.5	29.2	-4.3	32.4	27.8	-4.6
	Heart Failure(I50)	33.5	29.2	-4.3	32.4	27.8	-4.6
	Ischemic Heart Disease (I20-I25)	33.5	29.2	-4.3	32.4	27.8	-4.6
15–34 years	Colorectal cancer(C20)	46.8	43.3	-3.5	40.1	36.6	-3.5
	Esophagus cancer(C15)	61.8	58.9	-2.9	56.0	52.7	-3.3
	Stomach/gastric cancer(C16)	25.1	23.0	-2.2	21.1	19.0	-2.1
	Hepatocellular cancer(C22)	53.2	50.2	-3.0	47.3	44.0	-3.3
	Larynx cancer(C32)	67.4	64.8	-2.6	62.1	59.1	-3.0
	Oral cancer(C00-C14)	67.4	64.8	-2.6	62.1	59.1	-3.0
	Pancreas cancer(C25)	31.4	28.7	-2.7	26.3	23.7	-2.6
	Prostate cancer(C61)	15.3	13.7	-1.6	-	-	-
	Lung cancer (C34)	33.7	30.7	-3.0	28.1	25.1	-3.0
	Type 2 diabetes (E10-E14)	27.7	25.1	-2.6	22.8	20.3	-2.5
	Stroke(I63)	43.2	39.8	-3.4	36.7	33.2	-3.5
	Heart Failure(I50)	43.2	39.8	-3.4	36.7	33.2	-3.5
	IHD(I20-I25)	43.2	39.8	-3.4	36.7	33.2	-3.5
35-54years	Colorectal cancer(C20)	43.2	39.7	-3.5	34.2	31.9	-2.3
	Esophagus cancer(C15)	58.7	55.7	-3.0	50.3	48.3	-2.0
	Stomach/gastric cancer(C16)	23.0	20.8	-2.2	17.7	16.4	-1.3
	Hepatocellular cancer(C22)	50.1	47.0	-3.1	41.8	39.7	-2.1
	Larynx cancer(C32)	64.6	61.8	-2.7	56.8	55.0	-1.8
	Oral cancer(C00-C14)	64.6	61.8	-2.7	56.8	55.0	-1.8
	Pancreas cancer(C25)	28.7	26.0	-2.7	22.1	20.5	-1.6
	Prostate cancer(C61)	13.7	12.1	-1.6	-	-	-
	Lung cancer (C34)	30.7	27.7	-3.0	23.4	21.6	-1.8
	Type 2 diabetes (E10-E14)	25.1	22.5	-2.6	18.9	17.4	-1.5
	Stroke(I63)	39.7	36.3	-3.4	31.1	28.9	-2.2
	Heart Failure(I50)	39.7	36.3	-3.4	31.1	28.9	-2
	Ischemic Heart Disease (I20-I25)	39.7	36.3	-3.4	31.1	28.9	-2.2
55–64 years	Colorectal cancer(C20)	38.7	36.1	-2.6	30.8	31.2	+0.3
	Esophagus cancer(C15)	54.7	52.3	-2.4	47.1	47.6	+0.5
	Stomach/gastric cancer(C16)	20.3	18.7	-1.5	15.8	16.0	+0.2
	Hepatocellular cancer(C22)	46.0	43.7	-2.4	38.6	39.0	+0.4
	Larynx cancer(C32)	60.9	58.7	-2.2	53.8	54.4	+0.6
	Oral cancer(C00-C14)	60.9	58.7	-2.2	53.8	54.4	+0.6
	Pancreas cancer(C25)	25.3	23.4	-1.9	19.7	19.9	+0.2
	Prostate cancer(C61)	11.7	10.7	-1.0	-	-	-
	Lung cancer (C34)	27.0	24.8	-2.2	20.8	21.0	+0.2
	Type 2 diabetes (E10-E14)	21.9	20.1	-1.8	16.8	16.9	+0.1
	Stroke(I63)	35.3	32.9	-2.4	27.9	28.2	+0.3
	Heart Failure(I50)	35.3	32.9	-2.4	27.9	28.2	+0.3
	Ischemic Heart Disease (I20-I25)	35.3	32.9	-2.4	27.9	28.2	+0.3
65–74 years	Colorectal cancer(C20)	35.9	37.3	+1.4	31.0	29.4	-1.6
	Esophagus cancer(C15)	52.2	53.3	+1.1	47.1	45.6	-1.5
	Stomach/gastric cancer(C16)	18.6	19.5	+0.9	15.9	15.0	-0.9
	Hepatocellular cancer(C22)	43.5	44.7	+1.2	38.7	37.2	-1.5
	Larynx cancer(C32)	58.6	59.6	+1.0	53.8	52.4	-1.4
	Oral cancer(C00-C14)	58.6	59.6	+1.0	53.8	52.4	-1.4
	Pancreas cancer(C25)	23.3	24.4	+1.1	19.9	18.7	-1.2
	Prostate cancer(C61)	10.6	11.2	+0.6	-	-	-
	Lung cancer (C34)	24.7	25.9	+1.2	21.0	19.7	-1.3
	Type 2 diabetes (E10-E14)	20.0	21.0	+1.0	16.9	15.8	-1.1
	Stroke(I63)	32.7	34.0	+1.3	28.1	26.6	-1.6
	Heart Failure(I50)	32.7	34.0	+1.3	28.1	26.6	-1.5
	Ischemic Heart Disease (I20-I25)	32.7	34.0	+1.3	28.1	26.6	-1.5
75+ years	Colorectal cancer(C20)	34.6	34.3	-0.3	28.2	30.7	+2.5
	Esophagus cancer(C15)	50.7	50.6	-0.1	43.8	46.9	+3.1
	Stomach/gastric cancer(C16)	17.9	17.7	-0.2	14.4	15.7	+1.3
	Hepatocellular cancer(C22)	42.1	41.9	-0.2	35.7	38.5	+2.8
	Larynx cancer(C32)	57.2	57.1	-0.1	50.6	53.7	+3.1
	Oral cancer(C00-C14)	57.2	57.1	-0.1	50.6	53.7	+3.1
	Pancreas cancer(C25)	22.4	22.2	-0.2	18.0	19.6	+1.6
	Prostate cancer(C61)	10.2	10.0	-0.2	-	-	-
	Lung cancer (C34)	23.8	23.5	-0.3	19.0	20.7	+1.7
	Type 2 diabetes (E10-E14)	19.2	18.9	-0.3	15.2	16.6	+1.4
	Stroke(I63)	31.5	31.2	-0.3	25.5	27.8	+2.3
	Heart Failure(I50)	31.5	31.2	-0.3	25.5	27.8	+2.3
	Ischemic Heart Disease (I20-I25)	31.5	31.2	-0.3	25.5	27.8	+2.3

*ICD-10, International Classification of Diseases, 10^th^ Revision

Table 4 shows the economic burden of unhealthy eating in 2004 and in 2015 and the change between years estimated in 2017 Canadian dollars. The overall economic burden of unhealthy eating in Canada was estimated to be $13.21 billion ($4.24 billion in direct health care costs, $8.97 billion in indirect costs) in 2004. In 2015, it was estimated to be $13.08 billion ($4.18 billion in direct health care costs, $8.89 billion in indirect costs). The estimated change in the overall economic burden between 2004 and 2015 was a decrease of $133 million ($12.1 million/year). The estimated economic burden is about twice as high for males compared to females; however, the overall economic burden for males decreased by $219 million and for females increased by $86 million between 2004 and 2015. The economic burden due to poor diet quality decreased in those under 65 years (-$333 million) and increased in those 65 years and older (+$ 200 million). The economic burden decreased for all diseases with exception of oral cancer, which increased by $15 million, stroke which increased by $22 million, and heart failure which increased by $21 million.

**Table 4:** Economic burden of unhealthy diet by chronic disease, gender and age group (in 2017 $ CAN) in Canada.

	Attributable costs in 2017 $ CAN						Changes 2015 vs 2004
	2004			2015			
	Direct	Indirect	Total	Direct	Indirect	Total	
**DISEASE (ICD-10)**							
Colorectal cancer(C20)	109 029 668	538 606 562	647 636 230	107 214 127	529 637 786	636 851 914	-10 784 316
Esophagus cancer(C15)	46 535 728	229 886 495	276 422 223	46 066 272	227 567 383	273 633 657	-2 788 566
stomach/gastric cancer(C16)	29 279 371	144 640 095	173 919 467	28 986 576	143 193 684	172 180 259	-1 739 208
Hepatocellular cancer(C22)	13 984 178	69 081 841	83 066 020	13 690 859	67 632 841	81 323 699	-1 742 321
Larynx cancer(C32)	34 245 916	169 174 824	203 420 740	33 972 017	167 821 764	201 793 781	-1 626 959
Oral cancer(C00-C14)	130 646 735	645 394 871	776 041 605	133 179 854	657 908 477	791 088 329	15 046 724
Pancreas cancer(C25)	73 198 999	361 603 055	434 802 054	72 929 263	360 270 561	433 199 823	-1 602 231
Prostate cancer(C61)	58 109 777	287 062 299	345 172 076	57 213 470	282 634 542	339 848 013	-5 324 063
Lung cancer (C34)	139 890 329	691 058 228	830 948 557	138 337 103	683 385 288	821 722 390	-9 226 167
Type 2 diabetes (E10-E14)	736 615 873	891 305 206	1 627 921 078	700 673 101	847 814 453	1 548 487 553	-79 433 525
Stroke(I63)	418 337 971	719 541 310	1 137 879 280	426 446 233	733 487 522	1 159 933 756	22 054 476
Heart Failure(I50)	466 812 796	802 918 010	1 269 730 807	474 620 236	816 346 806	1 290 967 042	21 236 235
Ischemic Heart Disease (I20-I25)	1 989 777 241	3 422 416 855	5 412 194 096	1 961 447 510	3 373 689 716	5 335 137 226	-77 056 870
**Age group**							
<15 years	15 660 895	28 136 790	43 797 685	13 472 001	24 535 613	38 007 614	-5 790 071
15–34 years	63 552 723	103 521 887	167 074 610	57 540 350	94 324 290	151 864 640	-15 209 970
35–54 years	736 993 367	1 398 889 539	2 135 882 906	674 024 835	1 284 894 880	1 958 919 714	-176 963 192
55–64 years	923 070 850	1 945 257 212	2 868 328 061	878 618 081	1 854 232 431	2 732 850 513	-135 477 549
65–74 years	1 080 673 531	2 340 889 315	3 421 562 847	1 090 851 811	2 366 372 315	3 457 224 126	35 661 280
75+ years	1 426 513 218	3 155 994 905	4 582 508 123	1 480 269 544	3 267 031 295	4 747 300 839	164 792 716
**Gender**							
Male	2 687 141 216	5 719 965 722	8 407 106 937	2 609 869 447	5 578 584 181	8 188 453 627	-218 653 311
Female	1 559 323 367	3 252 723 929	4 812 047 295	1 584 907 17	3 312 806 643	4 897 713 815	85 666 521
**Total**	4 246 464 583	8 972 689 651	13 219 154 232	4 194 776 620	8 891 390 823	13 086 167 443	-132 986 787

Results of sensitivity analyses are presented in S4 and S5 Tables. When we used the upper and lower confidence interval boundaries of the relative risks and re-calculated PAFs estimates, the economic burden ranged, respectively, from $10.22 billion (-23%) to $16.38 billion (+23.9%) in 2004 and from $9.19 billion (-30%) to $16.23 billion (+24%) in 2015.

## 4. Discussion

Based on 24-hour recall data of large representative samples of the Canadian population, and using administrative health data, we estimated the economic burden of unhealthy eating to be $13.21 billion in 2004 and $13.08 billion in 2015. We observed that modest temporal improvements in diet quality resulted in a net decrease of $130 million in economic burden between 2004 and 2015, or $ 12 million yearly. This modest improvement coincides with the implementation period of the Integrated Pan-Canadian Healthy Living Strategy [42] that include healthy eating targeting to increase the percentage of Canadians that eat healthy with 20 per cent by 2015. We recommend evaluative research to examine whether the observed decline in economic burden is attributable to this strategy. Moreover, our cost estimates denote that the economic burden of unhealthy eating remains high in Canada considering the relatively small improvements in diet quality and the associated economic burden at the population level. Our study aimed at providing decision makers and health policy planners in Canada with the current picture of the global economic burden associated to poor diet quality. This could guide the elaboration of future policy and intervention options that could be implemented to decrease the economic burden of chronic diseases attributed to poor diet quality.

Considering that previous economic burden studies on unhealthy eating in Canada have not focused on the overall diet quality or otherwise used dietary quality indices, comparisons across studies are limited. However, we would like to mention that Lieffers et al. (2018) [26] have estimated the economic burden of unhealthy eating defined as not meeting established recommendations for eight foods in Canada to be $13.8 billion in 2004 which is close to our estimates, with minor differences which may be attributed to the fact that Lieffers et al (2018) [26] included some additional chronic diseases (e.g., kidney cancer, leukemia, hemorrhagic stroke, chronic renal disease) for which evidence of causality is not fully established and for which the relationship with the HEI-2010 scores is not quantified.

We observed that the proportion of unhealthy eating is higher in males compared to females, resulting in the associated economic burden being twice as high in males compared to females. Our findings are compatible of those of Imamura et al. (2015) who assessed the dietary quality among men and women in 187 countries, including Canada, in 1990 and 2010 and found that better diets are observed for women compared with men [43]. Although the economic burden of unhealthy eating observed in our study was high in man compared to women, this burden among man decreased between 2004 and 2015 (-$219 million) due to an overall improvement in diet quality, especially among those eating a diet of very poor quality (quintile 1 and quintile 2). On the contrary, despite an overall improvement of the diet quality of women between 2004 and 2015 in most age categories, the economic burden of unhealthy eating did increase by $ 86 million in this group. This finding was due to a substantial increase in the proportion of women 55 years and older eating a diet of poor quality (quintile 1) which is also the age group for whom the risk of disease increases.

The economic burden of unhealthy eating decreased by $ 333 million in people under the age of 65 years between 2004 and 2015 because of an overall improvement in the quality of their diet particularly among those with a very poor diet quality (i.e., quintile 1 and quintile 2). Contrarily, among people over the age of 65 years, the economic burden increased by $ 200 million between 2004 and 2015. This may be for a large part due to a large proportion of females over the age of 75 years with very poor quality diets. This result echoes findings from a study conducted by Lamage-Morin & Garriguet [44] who reported that the risk of poor nutrition status is common among seniors living in private households in Canada. Indeed, based on the results of the 2008/2009 CCHS, they reported that 34% of Canadians aged 65 years and older were at the risk of poor nutrition status with females more likely to be at risk compared to males. Disability, poor oral health, medication, living alone, low social support, infrequent social participation, not driving on a regular basis, lower income and education were the main characteristics associated with poor nutrition status in older adults [44]. In light of the aging of the population and the increase in the number of seniors, which could reach 25% of the total population in Canada [45], our observations further suggest policy and decision makers should consider the development of strategies to improve the diet quality in this age group.

We observed that among people below the age of 35 years, diet quality and its economic burden improved between 2004 and 2015. As these improvements coincide with the implementation of various programs promoting physical activity and healthy eating across Canada, especially in schools [46], it is important to examine whether this result could be attributable to the implementation of these programs.

The present study has several strengths. To our knowledge, this is the first study in Canada that has estimated the economic burden of unhealthy eating using a measure which assesses whole diet quality [16]. Various others studies had considered single dietary components [18, 19, 21, 23, 26]. To our knowledge, this is also the first study that has assessed temporal changes in the economic burden of the consumption of poor quality diets using the 2004 and 2015 CCHS Nutrition data. Another strength is that diet quality was assessed using 24-hour dietary recalls from large, representative samples of the Canadian population. In addition, we considered the relative risks retrieved in meta-analyses as random parameters and calculated population-attributable fraction (PAFs) using values from relative-risk distributions estimated by Monte Carlo simulations [39]. This helped us to take into account some of the variability of the HEI-2010 distribution scores across various populations. The HEI-2010 scores and health outcomes included in meta-analyses came from international studies. We acknowledge that we do not know if the Canadian adaption of HEI-2010 perfectly applies to these estimates. Other limitations may include the following: our study only considered chronic diseases for which an association with the HEI-2010 had been studied and for which evidence about this association was established. This may have led to an underestimation of the economic burden of unhealthy eating. In addition, the underestimation may also be due to the fact that we restricted our cost estimation to costs associated with treatment and management of chronic diseases without including costs related to other health professional (other than physicians) expenditures and other health care expenditures which have been included in other related Canadian studies [23, 47]. Indeed, if we make similar assumptions of including other costs not allocated in the EBIC tool as done by Krueger et al. (2015) [47] in their estimation of economic burden attributable to physical inactivity ($10.8 billion), excess body weight ($23.3 billion) and tobacco smoking ($18.7 billion), unhealthy eating would cost around 50% higher (i.e. $ 19.7 billion) exceeding the costs of tobacco smoking.

Our study may also be criticized for having included children and teenagers with regard to the estimation of economic burden of chronic diseases associated to poor diet quality in this age category. However, we note that other Canadian studies did include them in their analyses [21, 23, 26, 47]. In addition, if we remove attributable costs for this age category ($ 44 million in 2004 and $38 million in 2015), the resulting economic burden (i.e. $ 13.175 billion in 2004 and $13.05 billion in 2015) remains close to our estimates. This limitation also applies to the inclusion of stomach cancer for which there is mixed/weak evidence. Exclusion of this condition would provide an estimated economic burden of **$** 13.05 billion and $12.91 billion, respectively in 2004 and 2015.

Another limitation concerns the assumption of a linear dose-response relationship between dietary exposures and disease risk in the calculation of relative risks for other quintiles. We believe that this assumption is justified as did Krueger et al. (2017) [23] who applied it to estimate the relative risks of other categories using the relative risks expressed in terms of highest (quintile 5) versus the lowest one (quintile 1). In addition, when we look at data from studies (e.g. Reedy et al. 2014 [48]) included in meta-analyses considered [5, 6], the dose response relationship is linear.

Another limitation relates to the cost-of illness approach used. Indeed, using a prevalence-based approach, we estimated the economic burden over specific year’s periods (2004 and 2015) instead of using an incident-based approach which estimates the lifetime costs of a condition from its onset until its disappearance [49, 50]. Given that a number of diseases (cancers, cardiovascular diseases and type 2 diabetes) included in our studies have long durations and therefore require long follow-up periods, the prevalence-based approach we used is well suited to estimate the economic burden. Nevertheless, we encourage researchers to consider an incidence-based approach to estimate the economic burden. In the Canadian context, this could be performed by the use of linked data, e.g., the CCHS-Nutrition and the Public Health Agency of Canada’s Canadian Chronic Disease Surveillance System (CCDSS) to estimate the incidence chronic diseases and the use of health services attributable to poor diet.

### Conclusion

The economic burden of consuming poor quality diets was estimated to be $13.21 billion and $13.08 billion (2017 Canadian dollars) respectively in 2004 and in 2015. This represents a decrease of $130 million or a yearly decrease of $ 12 million. Despite an overall decrease in the economic burden associated with unhealthy eating, the economic burden increased among the elderly, and more among females than among males. Policy and decision makers are especially encouraged to develop nutrition programs and policies targeting the elderly in addition to those targeting young people to prevent chronic diseases and to reduce health care costs.

## Supporting information

S1 Table Relative risks* for chronic diseases used in analyses. (DOCX)

S2 Table Estimated direct health care costs (2017 $CAN) associated with chronic diseases by sex and age group in Canada. (DOCX)

S3 Table Ratios of indirect to direct costs calculated from Economic Burden of Illness in Canada, 1998 used in analyses. (DOCX)

S4 Table HEI-C 2010 mean scores (%) adjusted according quintile scores by survey year (2004, 2015). (DOCX)

S5 Table Economic burden of consuming poor quality diets in Canada by survey year, chronic disease, sex and age group (in 2017 $ CAN) using relative risk CI 95% low bounding values. (DOCX)

S6 Table Economic burden of consuming poor quality diets by survey year, chronic disease, sex and age group (in 2017 $ CAN) in Canada using relative risks CI 95% upper bounding values. (DOCX)
