# Supplementary material for: Temporal changes in diet quality and the associated economic burden in Canada
Source: PLoS One. 2018 Nov 8;13(11):e0206877. doi: 10.1371/journal.pone.0206877 (PMC6224068; doi:10.1371/journal.pone.0206877)
Supplement: S1 Table — (DOCX) [file pone.0206877.s001.docx]

**S1 Table. Relative risks* for chronic diseases used in analyses**

| **Chronic disease (ICD-10 Code)** | **RR** | **95% CI** | |
| --- | --- | --- | --- |
|  |  | **Lower level** | **Upper level** |
| Colorectal cancer(C20) | 0.77 | 0.73 | 0.81 |
| Esophagus cancer(C15) | 0.66 | 0.46 | 0.94 |
| Stomach/gastric cancer(C16) | 0.90 | 0.72 | 1.12 |
| Hepatocellular cancer(C22) | 0.72 | 0.53 | 0.98 |
| Larynx cancer(C32) | 0.61 | 0.40 | 0.94 |
| Oral cancer(C00-C14) | 0.61 | 0.40 | 0.94 |
| Pancreas cancer(C25) | 0.85 | 0.74 | 0.98 |
| Prostate cancer(C61) | 0.93 | 0.89 | 0.97 |
| Lung cancer (C34) | 0.84 | 0.81 | 0.87 |
| Type 2 diabetes (E10-E14) | 0.87 | 0.82 | 0.93 |
| Stroke(I63) | 0.79 | 0.77 | 0.82 |
| Heart Failure(I50) | 0.79 | 0.77 | 0.82 |
| Ischemic Heart Disease (I20-I25) | 0.79 | 0.77 | 0.82 |

**Sources:** Schwingshackl L &Hoffmann G (2015)[1] et al. 2015 and Schwingshackl L, Bogensberger B & Hoffmann G (2018) [2]

1. Schwingshackl L, Hoffmann G. *Diet quality as assessed by the Healthy Eating Index, the Alternate Healthy Eating Index, the Dietary Approaches to Stop Hypertension score, and health outcomes: a systematic review and meta-analysis of cohort studies.* J Acad Nutr Diet, 2015. **115**(5): 780-800.

2. Schwingshackl L, Bogensberger B, Hoffmann G, *Diet Quality as Assessed by the Healthy Eating Index, Alternate Healthy Eating Index, Dietary Approaches to Stop Hypertension Score, and Health Outcomes: An Updated Systematic Review and Meta-Analysis of Cohort Studies.* J Acad Nutr Diet, 2018. **118**(1): 74-100.
