# Supplementary material for: Temporal changes in diet quality and the associated economic burden in Canada
Source: PLoS One. 2018 Nov 8;13(11):e0206877. doi: 10.1371/journal.pone.0206877 (PMC6224068; doi:10.1371/journal.pone.0206877)
Supplement: S2 Table — (DOCX) [file pone.0206877.s002.docx]

**S2 Table. Estimated direct health care costs (2017 $CAN) associated with chronic diseases by sex and age group in Canada**

|  | **0-14 years** | **15-34 years** | **35-54 years** | **55-64 years** | **65-74 years** | **75+ years** |
| --- | --- | --- | --- | --- | --- | --- |
| **Male** | | | | | | |
| Colorectal Cancer | 22,353.63 | 859,413.66 | 33,842,161.11 | 51,623,400.49 | 55,288,798.47 | 50,118,774.84 |
| Esophagus Cancer | 9,222.82 | 324,489.85 | 9,090,073.24 | 21,307,147.10 | 19,885,908.73 | 17,858,783.95 |
| Gastric Cancer | 8,441.23 | 839,318.00 | 12,629,530.92 | 17,728,067.09 | 27,434,930.95 | 34,071,480.77 |
| Hepatocellular Cancer | 635,692.92 | 277,159.11 | 4,157,793.34 | 7,605,859.63 | 7,307,760.56 | 4,961,632.36 |
| Larynx Cancer | 33,452.28 | 68,509.42 | 5,593,620.36 | 13,175,926.23 | 16,875,223.89 | 11,498,702.08 |
| Oral Cancer | 1,321,948.38 | 1,591,104.87 | 18,432,696.86 | 26,915,713.30 | 19,989,642.14 | 19,392,408.63 |
| Pancreas Cancer | 10,004.42 | 245,235.73 | 13,978,670.20 | 26,778,749.68 | 80,211,266.20 | 136,209,426.47 |
| Prostate Cancer | 83,209.25 | 142,044.99 | 28,371,010.45 | 109,318,949.66 | 175,865,507.91 | 222,949,685.89 |
| Lung Cancer | 57,782.61 | 669,747.52 | 24,433,556.95 | 69,156,626.10 | 114,376,363.58 | 103,159,181.88 |
| Type 2 diabetes | 30,550,909.32 | 77,618,401.81 | 730,129,821.27 | 513,466,853.52 | 454,017,165.62 | 325,776,901.91 |
| Stroke | 5,093,474 | 4,342,777 | 50,206,161 | 127,748,666 | 155,809,503 | 272,349,419 |
| Heart Failure | 2,799,512 | 3,501,459 | 37,233,307 | $ 103,754,770 | 188,499,238 | 452,863,318 |
| Ischemic Heart Disease | 690,129 | 15,035,687 | 687,461,966 | 1,122,554,668 | 1,214,111,327 | 1,073,730,952 |
| **Female** | | | | | | |
| Colorectal Cancer | 33,764.92 | 1,055,195.85 | 17,042,465.32 | 25,834,166.05 | 35,694,429.72 | 41,136,729.92 |
| Esophagus Cancer | 5,627.49 | 167,459.94 | 2,428,908.19 | 5,393,072.04 | 5,858,340.79 | 7,510,121.75 |
| Gastric Cancer | 20,379.74 | 1,374,712.21 | 8,769,960.67 | 21,409,162.20 | 14,636,075.78 | 26,268,650.76 |
| Hepatocellular Cancer | 997,116.66 | 59,178.92 | 835,896.13 | 1,238,149.86 | 1,653,789.71 | 2,184,662.34 |
| Larynx Cancer | 38,927.40 | 172,829.29 | 1,323,453.24 | 3,523,889.71 | 3,920,205.84 | 2,362,814.65 |
| Oral Cancer | 317,862.04 | 1,491,015.14 | 7,613,084.89 | 9,604,337.20 | 9,969,898.15 | 12,113,459.92 |
| Pancreas Cancer | 43,002.06 | 685,388.25 | 7,164,607.73 | 13,746,285.78 | 19,027,045.76 | 26,892,417.73 |
| Prostate Cancer | - | - |  | - | - | - |
| Lung Cancer | 186,170.69 | 1,036,476.10 | 41,963,209.09 | 65,610,374.84 | 99,129,785.04 | 84,802,986.63 |
| Type 2 diabetes | 14,998,536.17 | 86,647,244.10 | 384,234,423.48 | 389,775,960.77 | 391,953,534.31 | 220,402,440.55 |
| Stroke | 4,558,846 | 13,651,452 | 102,517,952 | 117,317,517 | 211,451,507 | 675,807,422 |
| Heart Failure | 4,700,461 | 2,366,559 | 24,741,694 | 50,752,842 | 128,082,994 | 582,331,778 |
| Ischemic Heart Disease | 426,379 | 7,362,457 | 196,702,971 | 383,210,915 | 555,287,655 | 951,522,789 |
