# Supplementary material for: Temporal changes in diet quality and the associated economic burden in Canada
Source: PLoS One. 2018 Nov 8;13(11):e0206877. doi: 10.1371/journal.pone.0206877 (PMC6224068; doi:10.1371/journal.pone.0206877)
Supplement: S3 Table — (DOCX) [file pone.0206877.s003.docx]

**S3 Table. Ratios of indirect to direct costs calculated from Economic Burden of Illness in Canada, 1998 used in analyses**

| Disease category | Ratio indirect costs/direct health care costs |
| --- | --- |
| Cancer | 4.87 |
| Cardiovascular diseases | 1.81 |
| Endocrine and related diseases (type 2 diabetes) | 1.19 |
