# Supplementary material for: Temporal changes in diet quality and the associated economic burden in Canada
Source: PLoS One. 2018 Nov 8;13(11):e0206877. doi: 10.1371/journal.pone.0206877 (PMC6224068; doi:10.1371/journal.pone.0206877)
Supplement: S4 Table — (DOCX) [file pone.0206877.s004.docx]

**S4 Table: HEI-C 2010 mean scores adjusted according to quintile scores by survey year (2004, 2015)**

|  | **Survey year 2004** | **Survey year 2015** |
| --- | --- | --- |
| Quintile 1 | 33.03 | 33.49 |
| Quintile 2 | 43.34 | 44.90 |
| Quintile 3 | 49.60 | 50.00 |
| Quintile 4 | 55.76 | 56.67 |
| Quintile 5 | 65.44 | 69.04* |

* indicates a significant variation compared to 2004 survey year
