# Supplementary material for: Temporal changes in diet quality and the associated economic burden in Canada
Source: PLoS One. 2018 Nov 8;13(11):e0206877. doi: 10.1371/journal.pone.0206877 (PMC6224068; doi:10.1371/journal.pone.0206877)
Supplement: S5 Table — (DOCX) [file pone.0206877.s005.docx]

**S5 Table: Economic burden of consuming poor quality diets in Canada by survey year, chronic disease, sex and age group (in 2017 $ CAN) using relative risk CI 95% low bounding values**

| SURVEY YEAR | Gender | DISEASE | Direct Health Care | Indirect | Total |
| --- | --- | --- | --- | --- | --- |
| 2004 | Male |  |  |  |  |
|  |  | Colorectal cancer(C20) | 84 592 065 | 417 884 801 | 502 476 866 |
|  |  | Esophagus cancer(C15) | 55 782 468 | 275 565 394 | 331 347 863 |
|  |  | stomach/gastric cancer(C16) | 41 767 562 | 206 331 756 | 248 099 318 |
|  |  | Hepatocellular cancer(C22) | 18 354 353 | 90 670 505 | 109 024 858 |
|  |  | Larynx cancer(C32) | 41 242 708 | 203 738 979 | 244 981 687 |
|  |  | Oral cancer(C00-C14) | 76 927 657 | 380 022 624 | 456 950 280 |
|  |  | Pancreas cancer(C25) | 104 680 641 | 517 122 366 | 621 803 007 |
|  |  | Prostate cancer(C61) | 92 015 436 | 454 556 256 | 546 571 692 |
|  |  | Lung cancer (C34) | 94 332 673 | 466 003 403 | 560 336 076 |
|  |  | Type 2 diabetes (E10-E14) | 659 297 570 | 797 750 060 | 1 457 047 631 |
|  |  | Stroke(I63) | 126 765 698 | 218 037 000 | 344 802 698 |
|  |  | Heart Failure(I50) | 283 276 775 | 487 236 053 | 770 512 828 |
|  |  | Ischemic Heart Disease(I20-I25) | 1 556 274 860 | 2 676 792 758 | 4 233 067 618 |
|  |  | **Total male** | **3 235 310 466** | **7 191 711 955** | **10 427 022 422** |
|  |  |  |  |  |  |
|  | Female | Colorectal cancer(C20) | 43 865 080 | 216 693 496 | 260 558 576 |
|  |  | Esophagus cancer(C15) | 16 013 869 | 79 108 512 | 95 122 381 |
|  |  | stomach/gastric cancer(C16) | 27 342 152 | 135 070 229 | 162 412 381 |
|  |  | Hepatocellular cancer(C22) | 4 624 405 | 22 844 563 | 27 468 968 |
|  |  | Larynx cancer(C32) | 9 386 642 | 46 370 013 | 55 756 655 |
|  |  | Oral cancer(C00-C14) | 121 782 191 | 601 604 022 | 723 386 213 |
|  |  | Pancreas cancer(C25) | 23 356 906 | 115 383 116 | 138 740 022 |
|  |  | Prostate cancer(C61) | 0 | 0 | 0 |
|  |  | Lung cancer (C34) | 72 941 031 | 360 328 692 | 433 269 723 |
|  |  | Type 2 diabetes (E10-E14) | 365 938 134 | 442 785 142 | 808 723 276 |
|  |  | Stroke(I63) | 334 178 815 | 574 787 563 | 908 966 378 |
|  |  | Heart Failure(I50) | 230 470 487 | 396 409 237 | 626 879 724 |
|  |  | Ischemic Heart Disease(I20-I25) | 628 440 635 | 1 080 917 892 | 1 709 358 526 |
|  |  | **Total female** | 1 878 340 347 | 4 072 302 476 | 5 950 642 823 |
|  |  |  |  |  |  |
|  |  | **Total male + female** |  |  |  |
|  |  |  |  |  |  |
| 2015 | Male | Colorectal cancer(C20) | 82 534 441 | 407 720 138 | 490 254 579 |
|  |  | Esophagus cancer(C15) | 55 360 208 | 273 479 428 | 328 839 636 |
|  |  | stomach/gastric cancer(C16) | 41 076 096 | 202 915 913 | 243 992 009 |
|  |  | Hepatocellular cancer(C22) | 18 098 262 | 89 405 417 | 107 503 679 |
|  |  | Larynx cancer(C32) | 41 074 856 | 202 909 789 | 243 984 645 |
|  |  | Oral cancer(C00-C14) | 76 360 940 | 377 223 042 | 453 583 982 |
|  |  | Pancreas cancer(C25) | 104 183 626 | 514 667 110 | 618 850 736 |
|  |  | Prostate cancer(C61) | 90 691 688 | 448 016 939 | 538 708 627 |
|  |  | Lung cancer (C34) | 93 044 092 | 459 637 815 | 552 681 907 |
|  |  | Type 2 diabetes (E10-E14) | 625 240 486 | 756 540 988 | 1 381 781 475 |
|  |  | Stroke(I63) | 125 000 152 | 215 000 261 | 340 000 412 |
|  |  | Heart Failure(I50) | 280 251 704 | 482 032 931 | 762 284 634 |
|  |  | Ischemic Heart Disease(I20-I25) | 1 515 986 673 | 2 607 497 077 | 4 123 483 749 |
|  |  | **Total male** | **3 148 903 224** | **7 037 046 848** | **10 185 950 070** |
|  |  |  |  |  |  |
|  | Female | Colorectal cancer(C20) | 44 122 572 | 217 965 506 | 262 088 078 |
|  |  | Esophagus cancer(C15) | 16 237 435 | 80 212 931 | 96 450 367 |
|  |  | stomach/gastric cancer(C16) | 27 717 165 | 136 922 796 | 164 639 961 |
|  |  | Hepatocellular cancer(C22) | 4 626 093 | 22 852 898 | 27 478 990 |
|  |  | Larynx cancer(C32) | 9 441 971 | 46 643 338 | 56 085 310 |
|  |  | Oral cancer(C00-C14) | 125 722 622 | 621 069 753 | 746 792 375 |
|  |  | Pancreas cancer(C25) | 23 656 061 | 116 860 939 | 140 517 000 |
|  |  | Prostate cancer(C61) | 0 | 0 | 0 |
|  |  | Lung cancer (C34) | 72 592 003 | 358 604 495 | 431 196 498 |
|  |  | Type 2 diabetes (E10-E14) | 355 101 427 | 429 672 727 | 784 774 154 |
|  |  | Stroke(I63) | 345 177 830 | 593 705 868 | 938 883 698 |
|  |  | Heart Failure(I50) | 242 349 036 | 416 840 342 | 659 189 378 |
|  |  | Ischemic Heart Disease(I20-I25) | 640 176 182 | 1 101 103 033 | 1 741 279 215 |
|  |  | **Total female** | **1 906 920 397** | **4 142 454 626** | **6 049 375 023** |
|  |  | **Total male + female** | **5 055 823 621** | **11 179 501 473** | **16 235 325 093** |
