# Supplementary material for: Temporal changes in diet quality and the associated economic burden in Canada
Source: PLoS One. 2018 Nov 8;13(11):e0206877. doi: 10.1371/journal.pone.0206877 (PMC6224068; doi:10.1371/journal.pone.0206877)
Supplement: S6 Table — (DOCX) [file pone.0206877.s006.docx]

**S6 Table: Economic burden of consuming poor quality diets by survey year, chronic disease, sex and age group (in 2017 $ CAN) in Canada using relative risks CI 95% upper bounding values**

| SURVEY YEAR | Gender | DISEASE | Direct Health Care | Indirect | Total |
| --- | --- | --- | --- | --- | --- |
| 2004 | Male |  |  |  |  |
|  |  | Colorectal cancer(C20) | 59 688 044 | 294 858 936 | 354 546 980 |
|  |  | Esophagus cancer(C15) | 6 617 803 | 32 691 949 | 39 309 752 |
|  |  | stomach/gastric cancer(C16) | 15 885 827 | 78 475 984 | 94 361 811 |
|  |  | Hepatocellular cancer(C22) | 811 257 | 4 007 609 | 4 818 866 |
|  |  | Larynx cancer(C32) | 4 528 983 | 22 373 177 | 26 902 160 |
|  |  | Oral cancer(C00-C14) | 8 717 253 | 43 063 229 | 51 780 481 |
|  |  | Pancreas cancer(C25) | 7 768 474 | 38 376 262 | 46 144 737 |
|  |  | Prostate cancer(C61) | 24 713 899 | 122 086 663 | 146 800 562 |
|  |  | Lung cancer (C34) | 64 153 530 | 316 918 437 | 381 071 967 |
|  |  | Type 2 diabetes (E10-E14) | 257 233 099 | 311 252 050 | 568 485 149 |
|  |  | Stroke(I63) | 98 881 568 | 170 076 298 | 268 957 866 |
|  |  | Heart Failure(I50) | 220 666 827 | 379 546 943 | 600 213 770 |
|  |  | Ischemic Heart Disease(I20-I25) | 1 556 274 860 | 2 676 792 758 | 4 233 067 618 |
|  |  | **Total male** | **2 325 941 424** | **4 490 520 295** | **6 816 461 719** |
|  |  |  |  |  |  |
|  | Female | Colorectal cancer(C20) | 29 998 215 | 148 191 184 | 178 189 399 |
|  |  | Esophagus cancer(C15) | 1 575 831 | 7 784 606 | 9 360 437 |
|  |  | stomach/gastric cancer(C16) | 9 787 552 | 48 350 508 | 58 138 060 |
|  |  | Hepatocellular cancer(C22) | 174 389 | 861 479 | 1 035 868 |
|  |  | Larynx cancer(C32) | 851 708 | 4 207 440 | 5 059 148 |
|  |  | Oral cancer(C00-C14) | 10 569 183 | 52 211 763 | 62 780 946 |
|  |  | Pancreas cancer(C25) | 1 623 682 | 8 020 988 | 9 644 670 |
|  |  | Prostate cancer(C61) | 0 | 0 | 0 |
|  |  | Lung cancer (C34) | 48 699 509 | 240 575 575 | 289 275 084 |
|  |  | Type 2 diabetes (E10-E14) | 136 694 308 | 165 400 113 | 302 094 420 |
|  |  | Stroke(I63) | 256 351 217 | 440 924 093 | 697 275 310 |
|  |  | Heart Failure(I50) | 176 552 874 | 303 670 943 | 480 223 817 |
|  |  | Ischemic Heart Disease(I20-I25) | 482 121 917 | 829 249 697 | 1 311 371 614 |
|  |  | **Total female** | **1 155 000 385** | **2 249 448 389** | **3 404 448 773** |
|  |  |  |  |  |  |
|  |  | **Total male + female** | **3 480 941 810** | **6 739 968 683** | **10 220 910 491** |
|  |  |  |  |  |  |
| 2015 | Male | Colorectal cancer(C20) | 57 889 921 | 285 976 211 | 343 866 132 |
|  |  | Esophagus cancer(C15) | 6 368 927 | 31 462 499 | 37 831 426 |
|  |  | stomach/gastric cancer(C16) | 15 468 946 | 76 416 594 | 91 885 540 |
|  |  | Hepatocellular cancer(C22) | 772 331 | 3 815 316 | 4 587 647 |
|  |  | Larynx cancer(C32) | 4 402 268 | 21 747 202 | 26 149 470 |
|  |  | Oral cancer(C00-C14) | 8 244 312 | 40 726 903 | 48 971 215 |
|  |  | Pancreas cancer(C25) | 7 689 416 | 37 985 716 | 45 675 132 |
|  |  | Prostate cancer(C61) | 24 298 698 | 120 035 568 | 144 334 266 |
|  |  | Lung cancer (C34) | 63 154 112 | 311 981 311 | 375 135 423 |
|  |  | Type 2 diabetes (E10-E14) | 240 465 669 | 290 963 460 | 531 429 129 |
|  |  | Stroke(I63) | 97 285 026 | 167 330 245 | 264 615 272 |
|  |  | Heart Failure(I50) | 217 889 203 | 374 769 429 | 592 658 633 |
|  |  | Ischemic Heart Disease(I20-I25) | 1 182 220 280 | 2 033 418 881 | 3 215 639 161 |
|  |  | **Total male** | **1 926 149 110** | **3 796 629 335** | **5 722 778 447** |
|  |  |  |  |  |  |
|  | Female | Colorectal cancer(C20) | 30 039 205 | 148 393 673 | 178 432 878 |
|  |  | Esophagus cancer(C15) | 1 574 193 | 7 776 515 | 9 350 708 |
|  |  | stomach/gastric cancer(C16) | 9 841 649 | 48 617 747 | 58 459 397 |
|  |  | Hepatocellular cancer(C22) | 168 009 | 829 964 | 997 973 |
|  |  | Larynx cancer(C32) | 834 803 | 4 123 929 | 4 958 733 |
|  |  | Oral cancer(C00-C14) | 11 205 549 | 55 355 412 | 66 560 961 |
|  |  | Pancreas cancer(C25) | 1 623 281 | 8 019 009 | 9 642 290 |
|  |  | Prostate cancer(C61) | 0 | 0 | 0 |
|  |  | Lung cancer (C34) | 48 269 847 | 238 453 046 | 286 722 893 |
|  |  | Type 2 diabetes (E10-E14) | 131 180 088 | 158 727 906 | 289 907 994 |
|  |  | Stroke(I63) | 264 412 892 | 454 790 173 | 719 203 065 |
|  |  | Heart Failure(I50) | 185 606 298 | 319 242 833 | 504 849 131 |
|  |  | Ischemic Heart Disease(I20-I25) | 490 139 905 | 843 040 636 | 1 333 180 541 |
|  |  | **Total female** | **1 174 895 721** | **2 287 370 844** | **3 462 266 564** |
|  |  | **Total male + female** | **3 101 044 830** | **6 084 000 179** | **9 185 045 011** |
